# Tetanus and Massive Injections of Serum in the Spinal Column

**Published:** 1918-01

**Authors:** 


### TETANUS

**Tetanus and Massive Injections of Serum in the Spinal Column.** By MM. PIGNOL, BRISSET, and LEMONNIER, reported by Ch. WALTHER. Trans. and Abs. from the *Bulletins et Mémoires de la Société de Chirurgie de Paris*. Oct. 12, 1915.

P., B., and L. have found injections of large quantities of serum into the spinal column the surest and most economical way of administering serum treatment. 200 cc. of serum suffice for this method, whereas a quantity of 760 cc. has been necessary in some cases of subcutaneous and intravenous injections. Immobilization in a slanting position with head lowered is an essential accompaniment of the treatment, and should be continued for a considerable period.

The authors insist upon the necessity of thorough surgical cleansing both to remove foreign matter and to provide complete ventilation of all parts of the wound. They have used the following cleansing solutions : permanganate, hydrogen peroxide, iodine, and chloride of zinc.

Most of their fatal cases were due to the fixation of the diaphragm and respiratory muscles, comparatively few to general intoxication.

Spinal injections usually lead to marked improvement within two or three days.

Out of 33 cases there were 6 deaths. 1 of these was due to an insufficient supply of serum. 3 were due to respiratory disorders.

